# Metformin improves intestinal ischemia-reperfusion injury by reducing the formation of mitochondrial associated endoplasmic reticulum membranes (MAMs) and inhibiting ferroptosis in intestinal cells

**DOI:** 10.3389/fphar.2025.1581085

**Published:** 2025-05-08

**Authors:** Xu Zhao, Likun Yan, Lifei Tian, Xiaolong Zhang, Ruiting Liu, Zeyu Li

**Affiliations:** Department of General Surgery, Shaanxi Provincial People’s Hospital, Xi’an, Shaanxi, China

**Keywords:** metformin, intestinal I/R injury, mitochondria-associated membranes, ferroptosis, ROS, reactive oxygen species

## Abstract

**Introduction:**

Intestinal ischemia-reperfusion (I/R) injury represents an inevitable and formidable postoperative challenge for all clinical surgeons. Ferroptosis has emerged as a crucial factor in the pathogenesis of intestinal I/R injury. Metformin, which is known to exhibit antiferroptotic properties, has elicited significant attention from both researchers and clinicians. This study was designed to comprehensively examine the protective effects of metformin against intestinal I/R injury and to elucidate the underlying potential mechanisms.

**Methods:**

To achieve this goal, both in vivo and in vitro models of I/R injury were established. For the in vivo experiments, metformin was administered via intraperitoneal injection at the onset of reperfusion.

**Results:**

The results from HE staining in the in vivo model, along with IF staining of tight junction proteins in the in vitro model, clearly demonstrated that metformin effectively mitigated the damage to the intestinal barrier following I/R injury. Additionally, metformin was shown to improve ROS levels and mitochondrial function in the context of I/R injury. Moreover, metfornin was observed to reduce the formation of mitochondria‐associated membranes (MAMs), which is a process that is intricately linked to the onset of ferroptosis. Significantly, Western blot analysis of key ferroptosis-related proteins, including GPX4, FTH1 and SLC7A11, indicated that metformin inhibited ferroptosis.

**Discussion:**

In conclusion, this study suggests that metformin exerts beneficial effects on intestinal I/R injury by suppressing MAM formation and ferroptosis, thereby highlighting its potential as a therapeutic agent for this challenging clinical condition.

## Introduction

Ischemia reperfusion (I/R) injury refers to tissue damage caused by the process of restoring blood flow via surgery or medication after the interruption of organ blood flow. Ischemia itself can damage cells, whereas reperfusion can exacerbate this damage, thus leading to a considerable release of inflammatory response factors and an increase in the occurrence of cell death, which ultimately results in multiple organ failure (Li et al., 2021; Deng et al., 2022). I/R injury can occur in tissues and organs such as the heart, brain, liver, kidney, and intestines. Among these organs, the intestines (which are sensitive organs that are susceptible to injury) can be damaged in various events and diseases, such as intestinal surgery, intestinal volvulus, mesenteric artery embolism, strangulated intestinal obstruction, necrotizing colitis, intestinal transplantation or incarcerated hernia (Jia et al., 2020). The mechanism of intestinal IR injury is complex and intricate. Although numerous studies have been conducted, the mechanism and connections that are involved in intestinal IR injury have not been fully elucidated. Therefore, further research is still needed to provide new directions and ideas for treatment.

The mitochondria and the endoplasmic reticulum (ER) are key structures that regulate many cellular functions, and the highly dynamic and tightly coupled parts of these structures are known as mitochondria-associated endoplasmic reticulum membranes (MAMs). MAMs play an important roles in regulating cellular function and are closely related to intracellular calcium homeostasis, the oxidative stress response, mitochondrial homeostasis, and cell apoptosis (Angebault et al., 2018). In recent years, the increased relevance of MAMs in I/R injury has been demonstrated.

Ferroptosis is a new type of iron-dependent programmed cell death (Jiang et al., 2021). Recent studies have demonstrated that ferroptosis plays a critical role in intestinal I/R injury. Deng et al. (2021) reported lower levels of total glutathione (GSH, an endogenous antioxidant) and a lower total GSH/GSSG ratio (ratio of reduce to oxidized glutathione as a marker of oxidative stress), however, they also reported significantly increased levels of malondialdehyde (MDA, a byproduct and biomarker of lipid peroxidation) and Fe2+ and decreased mRNA and protein levels of the ferroptosis negative regulators Fth1 and Gpx4 in mice with intestinal I/R and ileal organoid H/R injury. However, after the administration of the ferroptosis inhibitor ferrostatin-1, these changes were reversed, thereby indicating that the inhibition of ferroptosis can reduce intestinal I/R injury *in vivo* and *in vitro*. Our previous research also confirmed that methane-rich saline improves intestinal IR injury by inhibiting ferroptosis (Fan et al., 2024). *Therefore, the inhibition of ferroptosis may be a new approach for treating intestinal I/R injury. Our previous research confirmed that the inhibition of MAM formation can reduce NLRP3 activation during I/R injury* (Li et al., 2024). Moreover, MAMs are key regulators of ferroptosis resulting in the exacerbation of acute lung injury (Li M. D. et al., 2022). Therefore, a reduction in the formation of MAMs may inhibit ferroptosis-induced intestinal I/R injury.

As a first-line medication for managing type 2 diabetes and metabolic syndrome, metformin is widely prescribed based on its insulin-sensitizing effects and favorable safety profile (Uto et al., 2019). In addition to its traditional hypoglycemic effect, it has been proven to provide protection against myocardial I/R injury (Tian et al., 2023). Nevertheless, the interplay between metformin and intestinal I/R injury (particularly its associated molecular signaling mechanisms) is not completely understood. Moreover, previous research has confirmed that metformin can target VDAC1, a calcium channel that is a component of the IP3R3-Grp75-VDAC1 complex that regulates calcium transfer from the ER to mitochondria. The component of the IP3R3-Grp75-VDAC1 complex is involved in the formation and stability of MAMs (Ko et al., 2024).

Therefore, we hypothesized that metformin improves intestinal I/R injury by inhibiting ferroptosis via the reduction of MAM formation. This study aimed to determine whether metformin affects intestinal I/R injury and to elucidate the potential mechanisms that are involved in this effect.

## Materials and methods

### Experimental animals

Male C57BL/6J mice (21–25 gB.W) were purchased from the Animal Center of Xi’an Jiaotong University. All of the mice were maintained in a standard environment at a temperature of 21°C–25°C, a humidity range of 40%–60%, and a 12 h light/dark cycle for 7 days before the experiments. All of the experimental procedures were approved and supervised by the Animal Ethics Committee of Xi’an Jiaotong University. The mice were randomly divided into three groups (n = 15): The sham group underwent a sham laparotomy operation. The IR group was intraperitoneally injected with normal saline at the beginning of reperfusion. The IR + Met group was intraperitoneally injected with metformin (TopScience, United States, 20 mg/kg) at the beginning of reperfusion. The mice were anesthetized with 1%–2% isoflurane. The superior mesenteric artery (SMA) was temporarily occluded with an atraumatic vascular clamp for 60 min. Afterwards the clamp was released to allow for reperfusion for 120 min. In total, intestinal and serum samples were collected from five mice, and the remaining 10 mice were fed for 72 h to calculate the survival rate.

### Experimental cell

The Caco-2 cell line (obtained from Shanghai Institutes of Biological Science) was utilized to establish I/R injury models. For hypoxia induction, the cells were maintained in a tri-gas incubator (Phcbi MCO-5 M, Japan) under hypoxic conditions (1% O_2_, 5% CO_2_, balanced with 94% N2) for 12 h. Subsequent reoxygenation was achieved by transferring the cells to a normoxic environment (5% O_2_, 5% CO_2_) for 2 h (Pereira et al., 2022). In the Met + IR group, the cells were pretreated with 2 mM metformin (Sigma-Aldrich) 30 min prior to the initiation of the IR protocol. All of the culturing procedures utilized DMEM supplemented with 10% fetal bovine serum (Gibco), which was maintained under standard conditions of temperature (37°C) and humidity.

### Tissue histological analysis

For histological evaluation, intestinal specimens were fixed in 4% neutral buffered formalin followed by paraffin embedding. Serial 4-μm sections were prepared using a microtome and subsequently subjected to hematoxylin-eosin (HE) staining. Histopathological scoring of intestinal damage was conducted according to established methodology from prior research (Jia et al., 2020). Histological injuries in the mucosa were evaluated by quantitative measured of tissue damage by a blinded observer.

### Elisa

The concentrations of proinflammatory cytokines (including tumor necrosis factor-α [TNF-α], interleukin-1β [IL-1β], and interleukin-6 [IL-6]) in intestinal tissues homogenates were quantitatively assessed using enzyme-linked immunosorbent assays (ELISAs). The serum levels of intestinal fatty acid-binding protein (I-FABP), which is a biomarker of enterocyte damage, were simultaneously measured through the same methodology. All of the analytical procedures were performed with commercial ELISA kits (Jiancheng Bioengineering Institute, Nanjing, China) following the manufacturer’s standardized protocols.

### Measurements of malondialdehyde, glutathione and iron

Intestinal tissue levels of glutathione (GSH), malondialdehyde (MDA), superoxide dismutase (SOD), and iron were assayed using a commercial biochemical kits (Nanjing Jiancheng, China).

### Determination of mitochondrial ROS

The MitoSOX Red Mitochondrial Superoxide Indicator Kit (Yeasen Biotechnology, China) and MitoTracker Green (Beyotime Biotechnology, China) were used to assess the levels of intracellular reactive oxygen species (ROS) in Caco-2 cell. Specifically, the MitoSOX Red reagent selectively targets mitochondria and fluoresces upon oxidation by superoxide anions, thereby providing a quantitative measure of mitochondrial ROS production. In addition, an 8-hydroxy-2′-deoxyguanosine (8-OHdG) detection kit (Beyotime Biotechnology, China) was used to evaluate DNA damage in intestinal tissues. Specifically, 8-OHdG is a well-established biomarker of oxidative DNA damage, and its measurement provides insights into the extent of DNA modification caused by oxidative stress in the intestinal epithelium. This approach allows for a comprehensive understanding of oxidative-stress-related cellular damage in the intestinal tissues.

### Transepithelial electrical resistance

As previously described (Xie et al., 2020), the integrity of the intestinal barrier was assessed via the measurement of transepithelial electrical resistance (TER) by using an Ussing chamber (EM-CSYS, PI, United States). This technique provides a quantitative method of assessing the functional integrity of the intestinal epithelial layer, as TER reflects the electrical resistance across the epithelium, which is closely associated with the integrity of tight junctions and overall barrier function.

### Cell viability and LDH assay

Cell viability of the Caco-2 cell was determined via a CCK-8 assay kit (Abcam, United States). Concurrently, lactate dehydrogenase (LDH) levels in Caco-2 cell were measured using a commercially available kit (Nanjing Jiancheng, China).

### Hydrogen peroxide quantification

The intracellular concentrations of hydrogen peroxide (H_2_O_2_) were precisely determined via a dedicated hydrogen peroxide assay kit. (Bio-Rad CA, United States).

### Complex activity measurement

Mitochondrial complex detection kits were used to determine the complex activity of the mitochondrial components. The protein concentration was determined via the BCA method, and a standard curve was plotted to ensure a consistent sample size and accuracy of activity measurement (Thermo Fisher Scientific). From each sample of whole cell lysate, 25 μg of protein was collected for activity detection of complexes I and V, and 70 μg of protein was collected for activity detection of complexes II + III. The absorbance was measured via a spectrophotometer, and the measurements were repeated three times for each result.

### Transmission electron microscopic analysis

TEM analysis was conducted in accordance with a previously reported protocol (Fan et al., 2024). Briefly, intestinal tissue samples were initially fixed in a 2.5% glutaraldehyde solution for 2 h. The samples were subsequently fixed in a 1% osmium tetroxide solution for 1 hour. Afterwards, the samples were dehydrated via a series of ethanol solutions of increasing concentrations. Finally, the dehydrated tissues were embedded in TAAB resin. For the assessment of mitochondria-endoplasmic reticulum contact points, ImageJ software was used following the methodology detailed in a previous study (Pereira et al., 2022). This approach allowed for a systematic and accurate quantification of these crucial cellular contacts, thus providing insights into the cellular ultrastructure and potential functional interactions between the mitochondria and endoplasmic reticulum.

### Immunofluorescence staining

Immunofluorescence staining was conducted following standard protocols (Li et al., 2019). Briefly, the cells and tissue sections were washed three times with PBS-T (PBS containing 0.25% Tween-20), followed by blocking with 5% goat serum. Primary antibodies against the following proteins were applied at a 1:200 dilution: occludin (Proteintech, China), ZO-1(Proteintech, China), IP3R (Abcam, United States), GRP75 (Proteintech, China), VDAC1 (Proteintech, China), SLC7A11 (Proteintech, China), and FTH1 (Proteintech, China), with overnight incubations at 4°C. The samples were then incubated with a secondary antibody (Proteintech, China; 1:100) for 1 h. Nuclei were counterstained with DAPI (Beyotime Biotechnology, China) prior to fluorescence microscopy analysis.

### Ca^2+^ determination

Caco-2 cells were seeded in 24-well plates. The calcium content in the mitochondria and endoplasmic reticulum was determined via the fluorescence intensity of Ca^2+^ by using Rhod-2AM (Yeasen Biotechnology, China) and Mag-fluo4 (Beyotime Biotechnology, China). MitoTracker Green (Beyotime Biotechnology, China) and ER-tracker Red (Beyotime Biotechnology, China) were used to locate the mitochondria or the endoplasmic reticulum in Caco-2 cells.

### Colocalization analysis of the ER, mitochondria and GPX4

Colocalization analysis of the mitochondria, ER and GPX4 was performed as previously described (Li J. et al., 2022). Caco-2 cells were incubated with the primary antibodies against GPX4 (1:200 Abcam, United States) and MitoTracker Green and ER-Tracker Red. The results were demonstrated using a confocal microscope.

### Immunohistochemistry

Immunohistochemistry was performed on mouse intestines as previously described (Li et al., 2019). The primary antibody anti-GPX4 (1:100; Abcam, United States) was incubated with the tissue sections at 4°C overnight, followed by incubation with secondary antibodies. The samples were subsequently stained with diaminobenzidine and counterstained with hematoxylin.

### Western blot analyses

Protein extraction in the Caco-2 cell was performed according to the manufacturer’s protocol. Following separation using 10%–25% gradient SDS-PAGE, the proteins were transferred to PVDF membranes and blocked with 8% skim milk. The membranes were probed overnight at 4°C with primary antibodies against GPX4 (1:1,000, Abcam), FTH1, and SLC7A11 (both 1:1,000, Proteintech). After incubation with species-matched HRP-conjugated secondary antibodies, protein expression was detected via chemiluminescence (ECL) and normalized to β-actin levels.

### Data analysis

Statistical analyses were performed using GraphPad Prism 6.0 (GraphPad Software, United States) and SPSS 22.0 (IBM Corp., United States), with quantitative data presented as the means ± SDs. Intergroup comparisons were conducted via unpaired Student's t tests for two independent cohorts, whereas multigroup comparisons were performed via one-way analysis of variance (ANOVA) with Tukey’s *post hoc* test. A probability threshold of P < 0.05 was predefined for establishing statistical significance in all of the experimental comparisons.

## Results

### Metformin alleviated the disruption of intestinal barrier function after I/R injury

**FIGURE 1:**
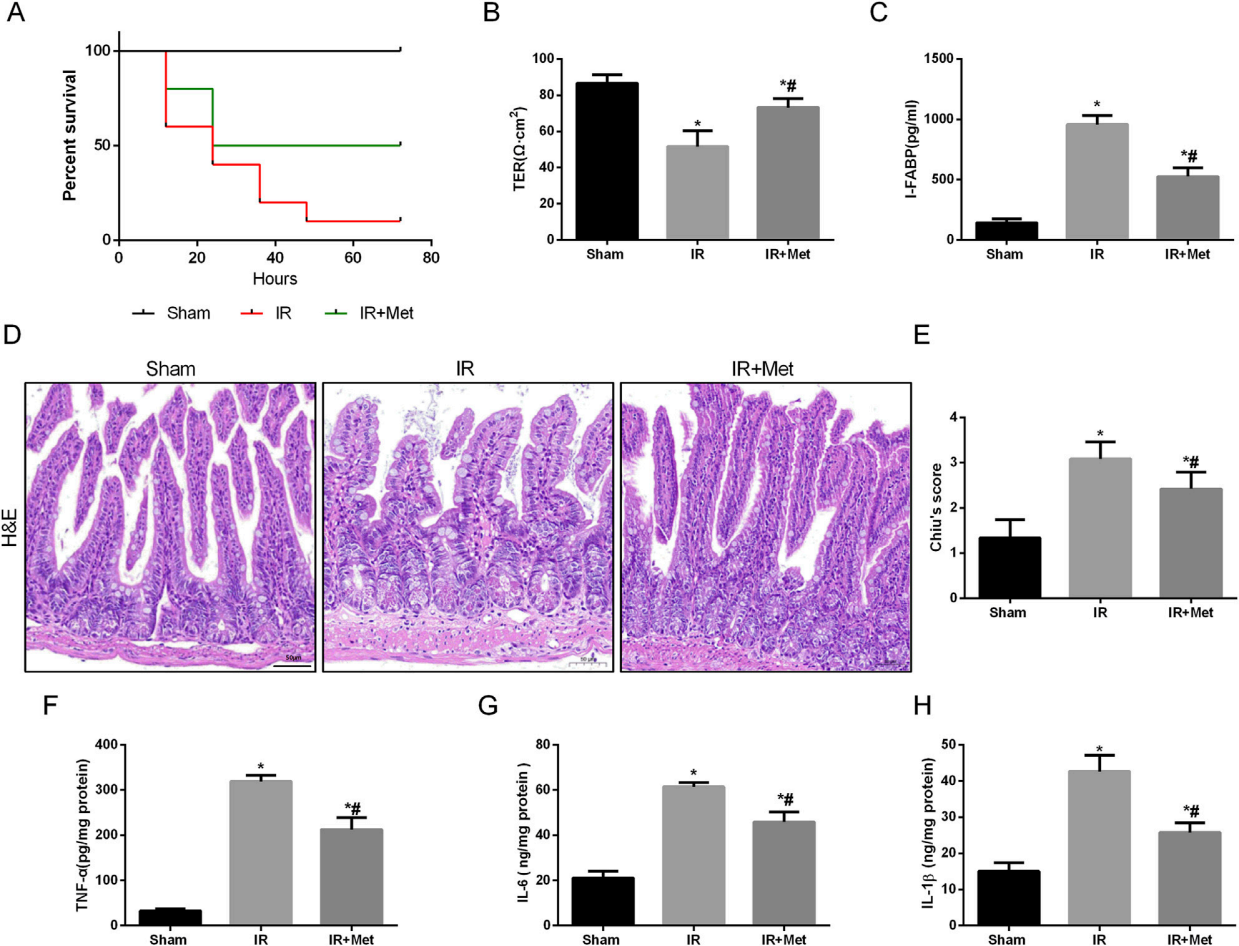
Metformin alleviated disruption of intestinal barrier function after I/R injury. Mice were subjected to I/R or sham surgery and divided into three groups: sham, IR and IR + Met group (n = 5). Intestines were collected after ischemia. **(A)** Survival rates were calculated in different groups (n = 10). **(B,C)** The integrity of the intestinal barrier was evaluated with TER and serum I-FABP levels. **(D,E)** Histopathological damage was estimated with HE staining (scale bars: 100 μm) and Chiu’s score. **(F–H)** The levels of TNF-α, IL-6 and IL-1β were evaluated by ELISA. The values are shown as the mean ± SD. ^*^p < 0.05, compared with the sham group, and ^#^p < 0.05 compared with the IR group.

**FIGURE 2:**
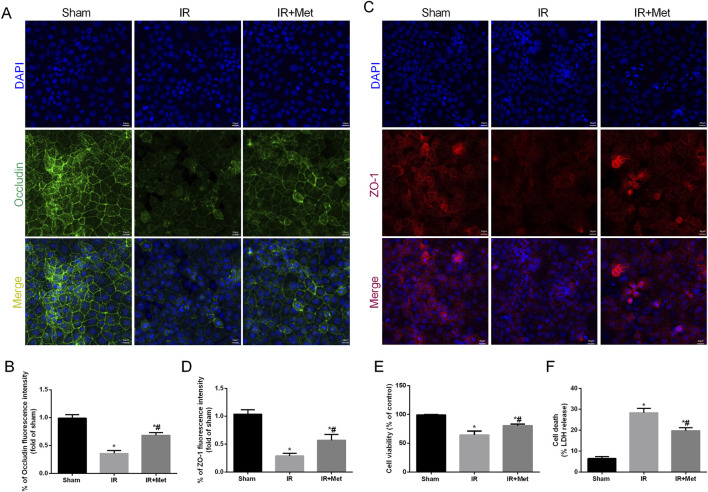
Metformin alleviated disruption of intestinal barrier function after I/R injury *in vitro*. The OGD/R model was established in Caco-2 cells. **(A–D)** The expression levels of ZO-1 and occludin were analyzed by IF staining (Scale bars: 20 μm). **(E)** The release levels of LDH were detected. **(F)** Cell viability was measured with a CCK-8 assay. The values are shown as the mean ± SD. ^*^p < 0.05, compared with the sham group, and ^#^p < 0.05 compared with the IR group.

To elucidate the impact of metformin on intestinal I/R injury, we evaluated the survival rate of mice following intestinal I/R. Our findings indicated that, in contrast to those mice in the sham group, the survival rate of the mice in the IR group was lower. Notably, treatment with metformin effectively increased the survival rate (Figure 1A). To assess the protective effect of metformin on intestinal barrier function during I/R injury, we subsequently measured the transepithelial electrical resistance (TER) in the three groups. Notably, compared with those in the sham group, the TER values in the I/R group tended to decrease. However, metformin treatment significantly mitigated this decline, thereby suggesting that metformin can improve intestinal barrier function during I/R injury (Figure 1B). Intestinal fatty acid-binding protein (I-FABP), which is a protein that is uniquely expressed in intestinal epithelial cells, is released extracellularly upon tissue damage; thus, it can serve as a biomarker for detecting intestinal injury during I/R injury (Voth et al., 2019). Our results revealed a significant increase in the level of I-FABP after I/R injury, thereby indicating evident intestinal disruption. This elevation was partially reversed by metformin treatment (Figure 1C). Furthermore, HE staining demonstrated significant architectural alterations in intestinal tissues, including disorganized arrangement of epithelial cells, blunted and atrophic villi accompanied by focal epithelial denudation and evident separation between the mucosal and submucosal layers, indicative of tissue edema and structural integrity compromise. Correspondingly, the histological score increased (Figures 1D,E). We subsequently detected inflammatory factors in intestinal tissues after I/R injury (Figures 1F–H). Compared with those in the sham group, the levels of TNF-α, IL-6, and IL-1β were significantly elevated in the IR group. Metformin treatment significantly ameliorated the levels of proinflammatory cytokines. In addition, we investigated the effect of metformin on intestinal cell function *in vitro* by using Caco-2 cells. The IF staining results indicated that the fluorescence intensities of the tight junction proteins occludin and ZO-1 were decreased after I/R injury (Figures 2A–D). Metformin treatment was able to increase the expression of occludin and ZO-1, which is consistent with the *in vivo* experimental results. Moreover, we discovered that metformin treatment could reduce LDH release and increase cell viability, thus further validating its protective effect during I/R injury (Figures 2E,F). Collectively, these results demonstrate that metformin can mitigate intestinal I/R injury.

### Metformin alleviated oxidative stress in I/R injury

**FIGURE 3:**
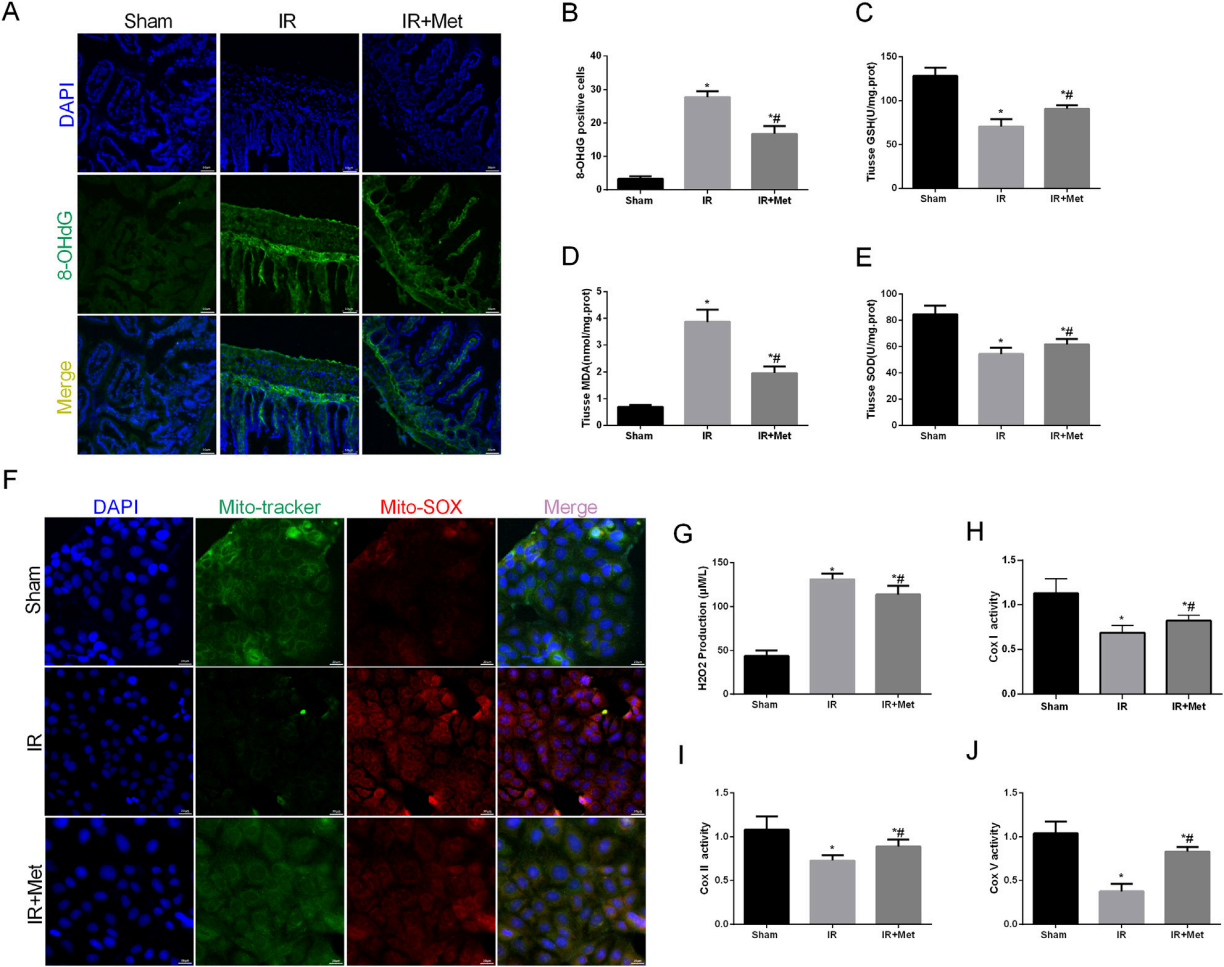
*Metformin alleviated oxidative stress in I/R injury*. The animal models were launched with a 60-min ischemia period as mentioned and the intestines were collected after ischemia. **(A,B)** Representative images of 8-OHdG staining of intestinal sections (Scale bars: 50 μm). The levels of **(C)** GSH, **(D)** MDA and **(E)** SOD were evaluated by ELISA. The OGD/R model was established in Caco-2 cells. **(F)** Representative ROS images of mitochondrion (Scale bars: 20 μm). **(G)** The levels of H2O2 were evaluated by ELISA. **(H–J)** The expression of Cox Ⅰ, Cox Ⅱ and Cox Ⅴ was analyzed by ELISA. The values are shown as the mean ± SD. ^*^p < 0.05, compared with the sham group, and ^#^p < 0.05 compared with the IR group.

Oxidative stress plays a crucial role in the disruption of the intestinal barrier following I/R injury. Therefore, we investigated the impact of metformin on counteracting the effects of reactive oxygen species (ROS) during I/R injury. Additionally, 8-OHdG serves as a biomarker for both endogenous and exogenous factors that induce DNA oxidative damage. It can be utilized to assess the extent of oxidative damage and repair, as well as the correlation between oxidative stress and DNA damage (Gauter-Fleckenstein et al., 2010). Fluorescence microscopy analysis revealed that the fluorescence intensity of 8-OHdG-positive cells was increased after I/R injury. However, treatment with metformin significantly attenuated this increase (Figures 3A,B). We subsequently measured the levels of GSH, MDA, and SOD in the intestines (Figures 3C–E). Compared with the sham group, I/R injury led to a decrease in the levels of GSH and SOD, whereas the level of MDA was increased. In contrast, metformin treatment significantly elevated the levels of GSH and SOD and decreased the level of MDA compared with those levels in the IR group. We further evaluated mitochondrial ROS in Caco-2 cells via IF staining (Figure 3F). I/R injury increased the fluorescence intensity of mitochondrial ROS; moreover, this effect was partially reversed by metformin treatment. Additionally, metformin inhibited ROS-induced oxidative stress, thereby reducing H_2_O_2_ production (Figure 3G). Intriguingly, we also observed that the expression of mitochondrial electron transport chain complexes, including cytochrome c oxidase subunit I (Cox-I), cytochrome c oxidase subunit II (Cox-II), and cytochrome c oxidase subunit V (Cox-V), was significantly decreased after I/R injury. Moreover, metformin treatment mitigated mitochondrial dysfunction (Figures 3H–J). In summary, these findings suggest that metformin mitigates intestinal I/R injury by enhancing mitochondrial function and reducing ROS-mediated damage.

### Metformin alleviated intestinal I/R injury by inhibiting MAM formation

**FIGURE 4:**
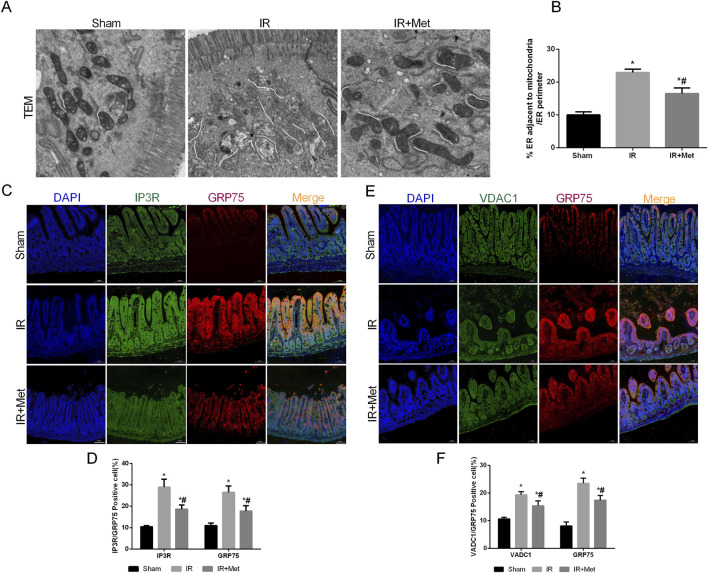
*Metformin alleviated intestinal I/R injury by inhibiting MAMs formation*. The animal models were launched with a 60-min ischemia period as mentioned. **(A,B)** Representative TEM images of MAMs. The white line illustrates quantitation of ER length adjacent to mitochondria normalized by total ER length (Scale bars: 500 nm). **(C,D)** The colocalization of IP3R and GRP75 was shown by immunofluorescence (Scale bars: 50 μm). **(E,F)** The colocalization of VDAC1 and GRP75 was shown by immunofluorescence (Scale bars: 50 μm). The values are shown as the mean ± SD. ^*^p < 0.05, compared with the sham group, and ^#^p < 0.05 compared with the IR group.

**FIGURE 5:**
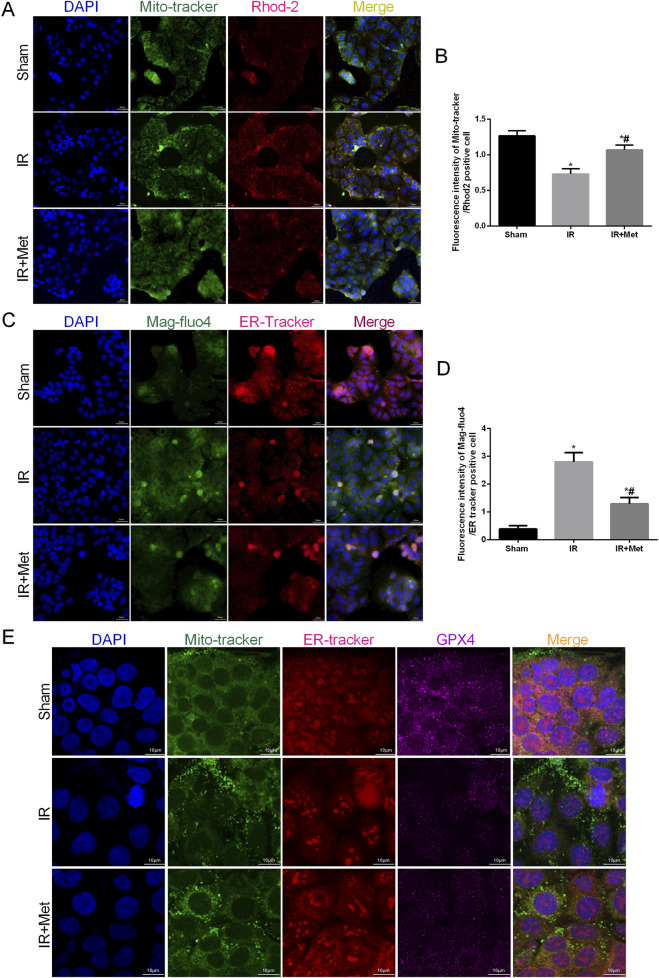
*Metformin maintained mitochondrial and endoplasmic reticulum calcium levels homeostasis.* The OGD/R model was established in Caco-2 cells. **(A,B)** The colocalization of Mito-tracker and Rhod-2 was shown by immunofluorescence (Scale bars: 20 μm). **(C,D)** The colocalization of Mag-fluo4r and ER-tracker was shown by immunofluorescence (Scale bars: 20 μm). **(E)** The colocalization of Mito-tracker, ER-tracker and GPX4 was shown by immunofluorescence confocal microscopy (Scale bars: 10 μm). The values are shown as the mean ± SD. ^#^p < 0.05, compared with the sham group, and ^$^p < 0.05 compared with the I/R group.

As pivotal areas of communication between the mitochondria and ER, mitochondria-associated endoplasmic reticulum membranes (MAMs) are essential for maintaining the functions of both the mitochondria and ER (Mao et al., 2022). Based on these findings, we aimed to evaluate the formation of MAMs following I/R injury and the impact of metformin on MAMs. This analysis was achieved by examining the physical interface between the ER and mitochondria using transmission electron microscopy (TEM) (Figures 4A,B). The results indicated that the physical interface between the ER and mitochondria was increased after I/R injury, whereas metformin treatment significantly attenuated the formation of MAMs. To further explore the effect of metformin on MAMs, we measured the expression of the IP3R/GRP75/VDAC1 signaling pathway. This pathway serves as a biomarker for MAMs and is a crucial channel for maintaining calcium homeostasis between the mitochondria and ER (Figures 4C–F). IF staining revealed that the fluorescence intensity of IP3R/GRP75/VDAC1 was increased after I/R injury. However, metformin treatment inhibited the expression of IP3R/GRP75/VDAC1, thereby suggesting that metformin could effectively reduce the formation of MAMs. Furthermore, we quantified the calcium levels in the mitochondria and ER of Caco-2 cells (Figures 5A–D). Based on the fluorescence intensities of Rhod-2a.m. and Mag-fluo4, compared with the sham group, I/R injury led to an accumulation of calcium in both the mitochondria and ER. In contrast, metformin treatment restored the calcium levels in the mitochondria and ER to levels that were significantly lower than those in the IR group. Given the established relationship between ferroptosis and MAMs, we investigated the effects of metformin on MAMs and glutathione peroxidase 4 (GPX4) using immunofluorescence confocal microscopy (Figure 5E). The findings revealed that I/R injury resulted in an increase in MAM formation and a concomitant decrease in GPX4 expression. Conversely, metformin treatment not only reduced the formation of MAMs but also promoted the expression of GPX4. Taken together, these results led us to hypothesize that metformin alleviates I/R injury by reducing the formation of MAMs and inhibiting ferroptosis.

### Metformin alleviated intestinal I/R injury by inhibiting ferroptosis

**FIGURE 6:**
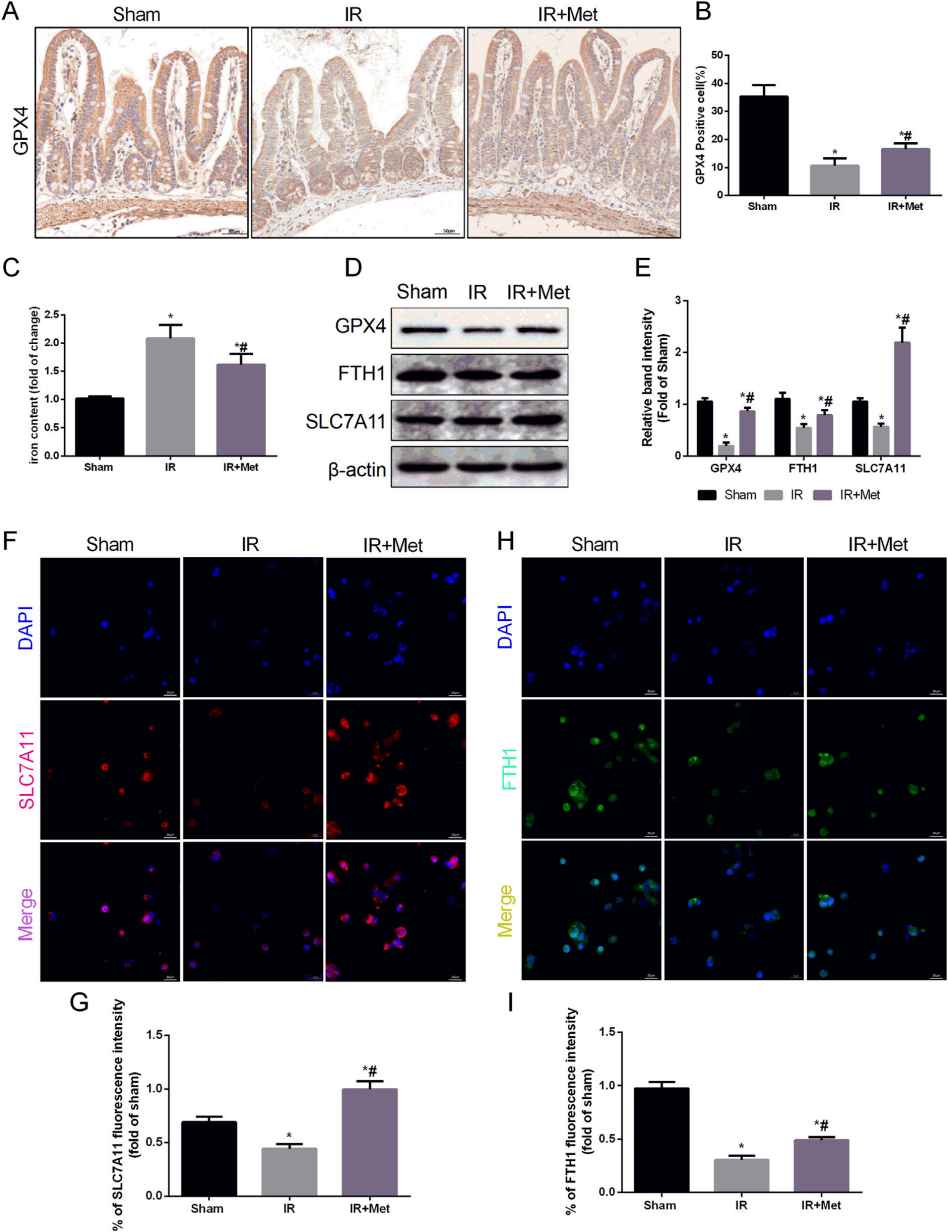
*Metformin alleviated intestinal I/R injury by inhibiting ferroptosis*. The animal models were launched with a 60-min ischemia period as mentioned and the intestines were collected after ischemia. **(A,B)** Immunohistochemical staining of GPX4 (Scale bars: 50 μm). **(C)** The levels of iron were evaluated by ELISA. The OGD/R model was established in Caco-2 cells. **(D,E)** The expression of GPX4, FTH1 and SLC7A11 was measured by Western blotting. **(F–I)** The expression levels of SLC7A11 and FTH1 were analyzed by IF staining (Scale bars: 20 μm). The values are shown as the mean ± SD. ^#^p < 0.05, compared with the sham group, and ^$^p < 0.05 compared with the I/R group.

To confirm that metformin can protect against intestinal I/R injury via the inhibition of GPX4-regulated ferroptosis, we utilized IHC staining to assess the expression of GPX4 following I/R injury (Figures 6A,B). Compared with that in the sham group, IHC staining demonstrated that the expression of GPX4 in the I/R group was decreased. Conversely, metformin treatment promoted the expression of GPX4, which aligned with the findings from the immunofluorescence confocal microscopy experiment in Caco-2 cells. Moreover, metformin was also observed to reduce iron ion levels (Figure 6C). We subsequently evaluated the expression levels of GPX4, ferritin heavy chain 1 (FTH1), and solute carrier family 7 member 11 (SLC7A11) via Western blot analysis (Figures 6D,E). The Western blot results indicated that the expression of GPX4, FTH1, and SLC7A11 in the IR group was lower than that in the sham group. However, metformin treatment reversed this trend. When considering the *in vitro* experiments, IF staining further confirmed the inhibitory effect of metformin on ferroptosis. This effect was evidenced by the decreased expression of FTH1 and SLC7A11 in Caco-2 cells (Figures 6F–I). In conclusion, metformin mitigates intestinal I/R injury-induced ferroptosis by decreasing the formation of MAMs, thereby alleviating damage to the intestinal barrier.

## Discussion

Intestinal ischemia-reperfusion (I/R) injury poses an inevitable and formidable postoperative challenge for all clinical surgeons (Gonzalez et al., 2015). From a clinical perspective, the prevention of I/R injury is extremely difficult, which is due to the fact that the majority of I/R injuries occur following emergency gastrointestinal surgeries, thereby resulting in unforeseen and rapidly progressing damage. Intestinal barrier dysfunction is one of the most prevalent types of damage caused by intestinal I/R injury. This dysfunction can trigger a severe inflammatory response and subsequent organ dysfunction (Carden and Granger, 2000). Consequently, there is an urgent need to develop diagnostic and treatment strategies for intestinal I/R injury. The protective effects of metformin on I/R injury in organs such as the heart and brain have been well established. However, current research regarding its role in intestinal I/R injury remains limited (Leech et al., 2020; Guo et al., 2023). Our study revealed that metformin can mitigate disruption of the intestinal barrier, increase the survival rate of mice, and attenuate the inflammatory response occurring as a result of I/R injury. Moreover, both *in vivo* and *in vitro* experiments demonstrated that metformin could inhibit ROS-induced damage in the intestines after I/R injury. Our previous research demonstrated that modulating the formation of MAMs is a viable approach for alleviating I/R injury (Li et al., 2024). Therefore, we further investigated whether the protective effect of metformin was associated with MAM formation. We discovered that metformin effectively decreased MAM formation during I/R injury. As specialized regions linking the mitochondria and ER, MAMs play crucial roles in various programmed cell death processes (Li M. D. et al., 2022; Verfaillie et al., 2012; Zhou et al., 2011). Our findings indicated that metformin treatment could inhibit the onset of ferroptosis during I/R injury. Based on these results, we postulated that metformin safeguards against intestinal I/R injury by reducing ferroptosis via the attenuation of MAM formation.

Metformin, which was originally employed for the treatment of type 2 diabetes, has been demonstrated to confer protection against a variety of diseases because of its antioxidant properties (Zhou et al., 2018; Foretz et al., 2014). Research has indicated that metformin can mitigate H/R-induced cardiomyocyte injury, thereby suggesting its potential as a therapeutic approach for myocardial I/R injury (Peng et al., 2021). Additionally, metformin has been shown to alleviate cerebral I/R injury by modulating the AMPK/ULK1/PINK1/Parkin pathway (Guo et al., 2023). However, the role of metformin in intestinal I/R injury remains inadequately explored. In the present study, metformin elicited robust protective effects during intestinal I/R injury. These effects were achieved via the upregulation of tight junction proteins, thereby preserving the integrity of the intestinal barrier structure. Moreover, metformin attenuated the inflammatory response by reducing the levels of proinflammatory cytokines such as TNF-α, IL-6, and IL-1β. In addition to the complex inflammatory cascade, ROS that are generated during reperfusion are also key mediators of I/R injury. The antioxidant capacity of metformin in I/R injury has been reported in numerous investigations (Calvert et al., 2008). Our study further corroborated these findings, thereby demonstrating that metformin augmented the levels of the antioxidant enzyme glutathione (GSH) *in vivo*, which aligns with the findings of previous studies. Additionally, metformin restored the levels of malondialdehyde (MDA) and superoxide dismutase (SOD) *in vivo*, which indirectly reflects the extent of tissue peroxidation.

As functional and physically contiguous interfaces between the mitochondria and ER, MAMs are pivotal in calcium signaling, lipid metabolism, and oxidative stress regulation (Zhou et al., 2022). Under stress conditions, ROS are produced and can accumulate. Excessive ROS often instigate oxidative stress, which correspondingly promotes MAM formation, thus ultimately resulting in mitochondrial dysfunction (Che et al., 2021). In our study, we found that metformin inhibited the expression of IP3R/GRP75/VDAC1. These findings suggest that metformin suppresses MAM formation following I/R injury. Concurrently, metformin decreased calcium levels in both the mitochondria and ER, thereby maintaining intracellular calcium homeostasis.

Ferroptosis, which is a novel form of programmable cell death, is recognized as being contingent upon intracellular iron overload and lipid peroxidation (Dixon et al., 2012). Recent investigations have revealed that ferroptosis is not only associated with I/R injury but also intricately linked to MAMs (Liu et al., 2024; Zhu et al., 2024). Thus, the inhibition of ferroptosis may represent a promising therapeutic approach for the treatment of intestinal I/R injury. In a previous study employing a myocardial I/R injury model, metformin was observed to mitigate ferroptosis in cardiomyocytes, thereby alleviating cardiac I/R injury. This effect was achieved via the regulation of the NOX4/AMPKα signaling pathway (Wu et al., 2023). Additionally, the formation of MAMs is considered to regulate the occurrence of ferroptosis (Lin et al., 2024). Our findings confirmed that metformin exerted a protective effect against intestinal I/R injury. Specifically, it inhibited ferroptosis by reducing the formation of MAMs.

## Conclusion

In summary, our study demonstrated that metformin mitigated intestinal I/R injury. The underlying mechanism of this effect was demonstrated to be associated with the attenuation of MAM formation and the inhibition of ferroptosis. We posit that metformin exhibits substantial therapeutic promise for the management of intestinal I/R injury.

## Data Availability

The raw data supporting the conclusions of this article will be made available by the authors, without undue reservation.
